# An optimistic future of C_4_ crop broomcorn millet (*Panicum miliaceum* L.) for food security under increasing atmospheric CO_2_ concentrations

**DOI:** 10.7717/peerj.14024

**Published:** 2022-09-07

**Authors:** Xinrui Shi, Jie Shen, Bingjie Niu, Shu Kee Lam, Yuzheng Zong, Dongsheng Zhang, Xingyu Hao, Ping Li

**Affiliations:** 1 Shanxi Agricultural University, Taigu, China; 2 Changzhi University, Changzhi, China; 3 University of Melbourne, Melbourne, Australia; 4 Ministerial and Provincial Co-Innovation Centre for Endemic Crops Production with High-quality and Effciency in Loess Plateau, Taigu, China

**Keywords:** Elevated CO_2_ concentrations, Broomcorn millet, Quality, Nutrients, Metabolites

## Abstract

Broomcorn millet, a C_4_ cereal, has better tolerance to environmental stresses. Although elevated atmospheric CO_2_ concentration has led to grain nutrition reduction in most staple crops, studies evaluating its effects on broomcorn millet are still scarce. The yield, nutritional quality and metabolites of broomcorn millet were investigated under ambient CO_2_ (*a*CO_2_, 400 µmol mol^–1^) and elevated CO_2_ (*e*CO_2_, *a*CO_2_+ 200 µmol mol^–1^) for three years using open-top chambers (OTC). The results showed that the yield of broomcorn millet was markedly increased under *e*CO_2_ compared with *a*CO_2_. On average, *e*CO_2_ significantly increased the concentration of Mg (27.3%), Mn (14.6%), and B (21.2%) over three years, whereas it did not affect the concentration of P, K, Fe, Ca, Cu or Zn. Protein content was significantly decreased, whereas starch and oil concentrations were not changed by *e*CO_2_. With the greater increase in grain yield, *e*CO_2_ induced increase in the grain accumulations of P (23.87%), K (29.5%), Mn (40.08%), Ca (22.58%), Mg (51.31%), Zn (40.95%), B (48.54%), starch (16.96%) and oil (28.37%) on average for three years. Flavonoids such as kaempferol, apigenin, eriodictyol, luteolin, and chrysoeriol were accumulated under *e*CO_2_. The reduction in L-glutamine and L-lysine metabolites, which were the most representative amino acid in grain proteins, led to a reduction of protein concentration under *e*CO_2_. Broomcorn millet has more desirable nutritional traits for combating hidden hunger. This may potentially be useful for breeding more nutritious plants in the era of climate change.

## Introduction

Globally average atmospheric CO_2_ concentration (CO_2_) has been increased from about 278 to 412 µmol mol^−1^ from pre-industrial period to 2020 (Friedlingstein et al., 2020). There is broad consensus that climate changes are likely to pose challenge to agricultural production and food security (IPCC, 2013; Kumar, 2016). Elevated CO_2_ (*e*CO_2_) decreased grain protein content of C_3_ crops such as rice and wheat by around 15% (Myers et al., 2014). The concentration of N, P and Zn was decreased by 6%, 5% and 10% under *e*CO_2_ compared with ambient CO_2_ (*a*CO_2_), respectively, irrespective of soil, crop and year (Jin, Armstrong & Tang, 2018). The micronutrients, such as S, Mg, Ca, Fe, Zn, Mn, and Cd in wheat grains, were markedly reduced under the *e*CO_2_ conditions (Myers et al., 2014; Myers et al., 2015; Beleggia et al., 2017). McGrath & Lobell (2013) also found that 12 out of 21 nutrients including macro- and micro-elements in crops were significantly reduced under *e*CO_2_. Al-Hadeethi et al. (2019) argued that major food crops including wheat, rice, field peas and corn had decreased protein, Zn and Fe concentrations when grown at *e*CO_2_ by meta-analysis using 542 experimental observations from 135 studies. Reduction in the Zn and Fe contents of the edible parts of C_3_ crops under *e*CO_2_ will likely aggravate the health problems associated with Zn and Fe deficiencies in regions where cereals-based diets are predominant. This would have adverse effects on public health. Malnutrition is a chronic problem in most of Asia exemplified by the more than two billion people who suffer from ‘hidden hunger’, which is defined as the insufficient intake or absorption of vitamins and minerals, such as vitamin A, Zn and Fe (Li et al., 2018). Christopher et al. (2018) demonstrated that *e*CO_2_-induced Zn and Fe deficiencies could result in an additional 125.8 million disability-adjusted life-years globally over the period 2015–2050, leading to a greater burden of infectious diseases, diarrhea and anemia. On the other hand, the grain quality and nutrient contents of C_4_ crops maize and sorghum seem to be less affected by *e*CO_2_ (Myers et al., 2014). Jobe et al. (2020) also pointed that *e*CO_2_ led to a penalty in the content of proteins and micronutrients in most staple crops, with the possible exception of C_4_ crops, due to their high productivity and adaptability to warm and dry climates and better water and N use efficiency than C_3_ crops.

Millets is the sixth most important cereal grains, sustaining more than one-third of the world’s population (Verma & Patel, 2012; Changmei & Dorothy, 2014). Millets including foxtail millet (*Setaria italica*), finger millet (*Eleusine coracana*), broomcorn millet (*Panicum miliaceum*), little millet (*Panicum sumatrense*), are superior to major cereals in terms of excellent agroecological traits, nutritional quality, and the potential to ensure the immediate demands of food security (Muthamilarasan & Prasad, 2021). Millets serve as a staple food in many African and Asian populations (Bandyopadhyay, Muthamilarasan & Prasad, 2017). Broomcorn millet (also named proso millet), a C_4_ crop, is one of the oldest cereals in the Old World. Archaeological evidence revealed that it was domesticated in China about 10,000 years ago (Lu et al., 2009). This cereal has diverse utilization in foods and as a forage plant (Bandyopadhyay, Muthamilarasan & Prasad, 2017; Sakamoto, 1987). Due to its high tolerance to poor soil, drought and high temperature, broomcorn millet is still serving as a major food crop in arid and semi-arid areas of China, and is also being extensively cultivated in arid regions of many other countries such as Eurasia, Oceania, North America, and more rarely in Africa (Habiyaremye et al., 2017). Broomcorn millet has a significant role in providing significant amounts of antioxidants and bioactive phytochemicals in the diet (Zhang, Liu & Wei, 2014). Kabog millet, an ecotype of broomcorn millet had higher total dietary fiber, total protein, total phenolic acids, tocopherols, and carotenoids content than white rice (Narciso & Nystrm, 2020). Wang et al. (2007) found accessions with protein content greater than 15% by screening 6,515 germplasm accessions from 14 provinces of China. Higher contents of certain (but not all) essential amino acids (especially leucine) that commonly found in wheat were showed for broomcorn millet (Kalinova & Moudry, 2006; Wiedemair et al., 2020). Similar to other cereal grains, lysine and the sulfur-containing methionine and cysteine represented limiting essential amino acids (Shen et al., 2018). Broomcorn millet has been identified as Future Smart Food (FSF) for Asia and hold great promise for development (Siddique, Li & Gruber, 2021). Our previous study revealed that *e*CO_2_ stimulated the yields of broomcorn millet on average by 25.5% in two years (Hao et al., 2017), and the yields of foxtail millet on average by 21.5% in two years (Li et al., 2019). However, the response of broomcorn millet grain nutrient concentrations and metabolites to *e*CO_2_ remains largely unknown.

Therefore, in this study, a pot experiment at the open-top chambers (OTC) was carried out to investigate the effects of *e*CO_2_ on the yield and grain quality of broomcorn millet. The objective of this study was to evaluate (1) how the yield and nutrient quality of broomcorn millet change under *e*CO_2_ scenario, and (2) whether *e*CO_2_ alters grain metabolites in broomcorn millet.

## Materials & Methods

### Site description

The pot experiments were carried out in 2013, 2015, 2016 at two OTC facilities, where located in Shanxi Agricultural University (37.42°N, 112.55°E), Taigu, Shanxi, China. The height and diameter of each OTC were 2.5 and 4.0 m respectively. The upper part of the OTC had a frustum of 0.5 m at 2.5 m height, to reduce the dilution of CO_2_ by air current inside the chamber and to keep it open and close to natural conditions. Detailed facility operational procedures can be found in Hao et al. (2017). The average temperature of each OTC during the broomcorn millet growing season were 23.4 °C, 23.1 °C and 22.9 °C in 2013, 2015 and 2016. The average relative humidity was 69.0%, 67.4% and 71.2% in 2013, 2015 and 2016 respectively.

### Experimental design

Different CO_2_ concentrations were set in the two OTC facilities. An *a* CO_2_ (approximately 400 µmol mol^−1^CO_2_) as the control (CK) treatment and another *e*CO_2_ (*a*CO_2_ + 200 µmol mol^−1^, approximately 600 µmol mol^−1^CO_2_ as EC treatment) were used from crop emergence to harvest.

HuachiRuan Red millet (Panicum miliaceum L.) from Huachi county, Gansu province, were sown on 13 June 2013, 17 June 2015 and 22 June 2016 in 40 cm × 60 cm pots (28 cm depth). The soil was obtained from a nearby cropland (0–20 cm surface soil). The soil type was clay loam, and the soil total N content, organic C content and pH value was 0.12%, 1.37% and 8.3 respectively. The soil was sieved, homogeneously mixed and packed in the pots. Five holes were made to drain away water in each pot bottom.

Fertilizers were applied at the elongation stage of millets at 1.7 g N and 1.15 g P_2_O_5_ per pot. Ten plants were grown in each pot and 10 pots were included in every chamber. Irrigation equivalent to 10–20 mm of rainfall was applied every 3–5 d to keep the soil water content in 60–80% of relative water content after seedling emergence. The water content was measured by wet sensor (KZSF, China).

### Harvesting and chemical analyses

At maturity, broomcorn millet plants were hand-harvested on 18 September 2013 (99 days after sowing), 25 September 2015 (100 days after sowing) and 1 October 2016 (103 days after sowing). The seeds were separated from the spikes after air drying, and air-dried at room temperature to constant weight, and then the yield was weighed. All of the seeds were ground into fine powder in preparation for chemical analyses.

The Kjeldahl method (China agricultural trade standard NY/T 3, 1982) was used to measure the protein concentration of seeds. 0.1 g of powdered seed was put into a Kjeldahl bottle for acidolysis and three mL concentrated sulfuric (18.4 mol L^−1^) acid was added. Then the liquid was heated by electric stove to keep it boiling for 1 h. 0.05 mol L^−1^ HCl was titrated until the color of liquid changed from blue–green to grayish purple. The crude protein concentration was calculated based on the dosage of HCl and a conversion factor of 6.25 was used.

The total oil concentration of seeds was evaluated by extraction with petroleum ether (60–80° C), with a soxhlet apparatus, and fat content was obtained by China agricultural trade standard NY/T 4 (1982). Total starch concentration was analyzed by enzyme hydrolysis methods (Mcleary, Solah & Gibson, 1994).

The seed was milled in a Willey-type mill. N was measured from the digestion of samples in H_2_SO_4_, distillation with the Kjeldahl distiller and titration in H_2_SO_4_ solution (Bataglia et al., 1983).

From the digestion of the samples in HNO_3_ and perchloric acid solution, P was determined from the phosphovanadomolybdic complex formed in the reaction of P with the solution of molybdovanadate at 450 nm in a 722S spectrophotometer (INESA, Shanghai, China) (Bataglia et al., 1983). 0.5 g of dried seed was digested in 10 mL of HNO_3_ and 2.5 mL HClO_4_ (v/v 4:1) acid for 24 h at room temperature until clear liquid was obtained; subsequently, the liquid was diluted to 25 mL. The concentration of K, Fe, Mn, Ca, Cu, Zn, Mg and B was analyzed following the procedure described in Zarcinas, Catwright & Spouncer (1987) by using inductively-coupled plasma atomic emission spectrometry (ICP-AES) (Optima 5300DV, PerkinElmer, Waltham, Massachusetts, USA). Grain mineral accumulation was calculated from the yield per m^2^ of plot surface area (g m^−2^) and grain mineral concentration.

### Metabolite extraction and metabolite profiling analysis

The seed was crushed using a mixer mill (MM 400, Retsch, Haan, Germany) with a zirconia bead for 1.5 min at 30 Hz. 100 mg powder was weighted and extracted overnight at 4 °C with 1.0 ml 70% aqueous methanol. The extracts were centrifugated at 10,000g for 10 min, then absorbed (CNWBOND Carbon-GCB SPE Cartridge, 250 mg, 3 ml; ANPEL, Shanghai, China) and filtrated (SCAA-104, 0.22 mm pore size; ANPEL, Shanghai, China) before LC-MS analysis. The sample extracts were analyzed for metabolites using an LC-ESI-MS/MS system (HPLC, Shim-pack UFLC SHIMADZU CBM30Asystem, Kyoto, Japan; MS, Applied Biosystems 6500 quadrupole-linear ion trap mass spectrometer Q TRAP, San Diego, California, USA) according to the method of Chen et al. (2013). Metabolite quantification was based on the MWDB (metware database) and public database of metabolite information. Metabolite intensity was conducted using multiple reaction monitoring mode (MRM) analysis (Fraga et al., 2010). The supervised multivariate method, orthogonal partial least squares discriminant analysis (OPLS-DA), was used to maximize the metabolome differences between the pair of samples. The relative importance of each metabolite to the OPLS-DA model was conformed using the parameter called variable importance in projection (VIP). Metabolites with VIP ≥1 and —log_2_ (fold change)—>1 were set as differential metabolites for group discrimination. For pathway annotation, all the metabolites were manually checked for their Kyoto Encyclopedia of Genes and Genomes (KEGG) name and number using KEGG database (Kanehisa et al., 2016), thereafter, classified in component classes and putative pathways.

### Statistical analysis

An analysis of variance (ANOVA) with two-way ANOVA by SAS System 8.1 (SAS Institute Inc., USA) was used to test the effects of (CO_2_) and year on yield, grain nutrients concentration, and grain accumulations of protein, starch, oil, and nutrients in broomcorn millet. Duncan’s multiple range tests at *P* = 0.05 were used to compare treatments.

## Results

### Response of yield, grain protein, starch and oil concentration to *e*CO_2_

**Figure 1:**
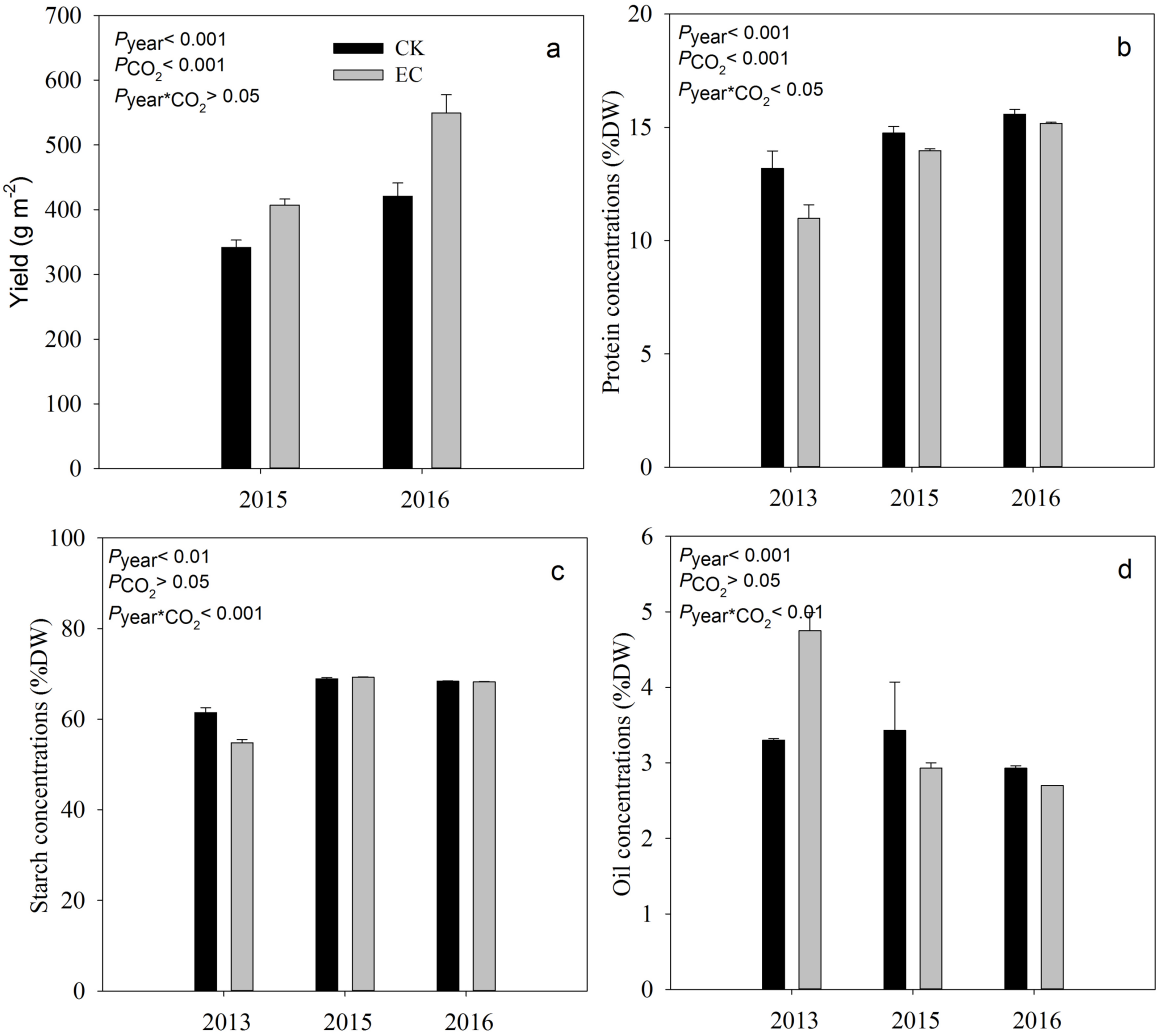
Effect of elevated CO_2_ on yield (a), grain protein (b), starch (c) and oil concentrations (d) in broomcorn millet. Each bar represents the standard error of the means (*n* = 10), *P*_year_, *P*_CO__2_ *P*_year * CO2_ represents the *P* values of the ANOVA results of year, CO_2_ concentration and their interaction effects on millet yield. CK- ambient atmospheric CO_2_ concentration; EC-elevated atmospheric CO_2_ concentration.

Compared with *a*CO_2_, *e*CO_2_ significantly increased the yield of broomcorn millet by 19.4% in 2015 and 29.9% in 2016 (*P* < .001) (Fig. 1). Grain protein concentration ranged from 10.98% to 15.58%. It was significantly decreased under *eCO*_2_ by 7.8%, averaged across three years (*P* < .001). Elevated CO_2_ had no significant effect on the concentration of starch and oil in broomcorn millet for three years (Fig. 1). A significant CO_2_ and year interaction was detected for the concentration of protein, starch, and oil.

### Response of grain mineral concentration to *e*CO_**2**_

Elevated CO_2_ significantly increased grain Mn, B, and Mg concentration across three years on average by 14.6%, 21.2%, and 27.3%, respectively (Table 1), while it had no significant effect on grain P, K, Fe, Ca, Cu or Zn concentration for three years. N concentration was significantly decreased by 7.8%, averaged across three years. Fe concentration was increased on average by 3.8% across three years. A significant CO_2_, year, and CO_2_x year interaction was detected for Mg concentration. Only significant CO_2_x year interaction was reported in K concentration which was attributable to the increased values in 2013 compared with 2015 and 2016 under *e*CO_2_.

### Response of the accumulations of grain nutrients, protein, starch, and oil in broomcorn millet to *e*CO_**2**_

Elevated CO_2_ significantly increased the grain mineral accumulations of P (+23.87%), K (+29.5%), Mn (+40.08%), Ca (+22.58%), Mg (+51.31%), Zn (+40.95%) and B (+48.54%) on average for three years (Table 2). Elevated CO_2_ significantly increased the grain accumulation of starch and oil for three years on average by 16.96% and 28.37%, respectively (Table 3). Grain protein accumulation ranged from 33.22 g m^−2^ to 83.02 g m^−2^. Elevated CO_2_ had no significant effect on the grain protein accumulations in 2013, and significantly increased it by 13.08% in 2015, 26.59% in 2016, respectively.

**Table 1:** Effect of elevated CO_2_ on grain mineral concentration in broomcorn millet seeds.

Year	Treatment	N	P	K	Fe	Mn	Ca	Mg	Cu	Zn	B
		(g kg^−1^)			(mg kg^−1^)						
2013	CK	21.1 ± 1.2	2.56 ± 0.16	2.10 ± 0.05	129.25 ± 18.52	7.18 ± 0.12	36.33 ± 0.88	649.17 ± 18.18	10.23 ± 1.03	58.85 ± 0.95	5.28 ± 0.72
	EC	17.6 ± 0.9	2.69 ± 0.15	2.85 ± 0.24	137.33 ± 23.84	10.17 ± 1.06	35.13 ± 1.46	1181.50 ± 212.28	13.70 ± 2.05	58.22 ± 1.83	5.55 ± 0.16
2015	CK	23.6 ± 0.5	2.39 ± 0.027	2.59 ± 0.06	91.52 ± 10.18	8.63 ± 0.10	32.7 ± 0.37	1215.25 ± 29.87	6.28 ± 0.25	47.78 ± 0.51	4.98 ± 0.45
	EC	22.3 ± 0.1	2.40 ± 0.068	2.42 ± 0.17	118.42 ± 21.22	9.25 ± 0.23	35.02 ± 1.69	1243.83 ± 66.40	8.38 ± 1.19	49.62 ± 1.08	6.45 ± 0.28
2016	CK	24.9 ± 0.3	2.38 ± 0.065	2.54 ± 0.08	125.18 ± 0.75	9.85 ± 0.28	32.95 ± 2.55	1176.33 ± 6.51	14.08 ± 4.47	59.07 ± 1.02	4.15 ± 0.56
	EC	24.3 ± 0.1	2.43 ± 0.083	2.39 ± 0.05	94.90 ± 4.24	10.00 ± 0.30	32.98 ± 0.97	1149.17 ± 42.97	9.37 ± 0.46	60.98 ± 1.13	5.47 ± 0.14
ANOVA	Year	***	ns	ns	ns	*	ns	**	ns	ns	ns
	CO_2_	***	ns	ns	ns	**	ns	*	ns	ns	**
	CO_2_× year	*	ns	**	ns	*	ns	**	ns	ns	ns

**Notes.**

Values are means ± standard error of variables across the three replicates, and the statistical significance level *P* values for the effects of CO_2_ treatment, year and their interaction.

CK ambient atmospheric CO_2_ concentration EC elevated atmospheric CO_2_ concentration ns means not significant (*P* > 0.05)

* significant at *P* < 0.05.

** significant at *P* < 0.01.

*** significant at *P* < 0.001.

**Table 2:** Effect of elevated CO_2_ on grain nutrient accumulations in broomcorn millet per m^2^ plot surface area.

Year	Treatment	N	P	K	Fe	Mn	Ca	Mg	Cu	Zn	B
		(g m^−2^)			(mg m^−2^)						
2013	CK	5.61 ± 0.25	0.68 ± 0.03	0.56 ± 0.01	34.47 ± 1.53	1.91 ± 0.05	9.68 ± 0.26	172.88 ± 3.64	2.73 ± 0.29	10.86 ± 4.54	1.41 ± 0.20
	EC	5.32 ± 0.29	0.81 ± 0.02	0.86 ± 0.04	41.54 ± 0.61	3.06 ± 0.23	10.62 ± 0.40	353.94 ± 51.82	4.12 ± 0.56	17.59 ± 0.28	1.68 ± 0.09
2015	CK	8.05 ± 0.24	0.82 ± 0.01	0.88 ± 0.02	31.22 ± 1.50	2.94 ± 0.03	11.15 ± 0.13	414.50 ± 10.19	2.14 ± 0.08	15.95 ± 0.17	1.69 ± 0.15
	EC	9.10 ± 0.07	0.98 ± 0.03	0.99 ± 0.07	48.24 ± 2.42	3.77 ± 0.09	14.26 ± 0.69	506.67 ± 27.05	3.41 ± 0.48	20.21 ± 0.44	2.63 ± 0.11
2016	CK	10.49 ± 0.17	1.00 ± 0.03	1.07 ± 0.03	52.98 ± 0.32	4.15 ± 0.12	13.87 ± 1.08	495.21 ± 2.74	5.93 ± 1.88	24.87 ± 0.43	1.75 ± 0.23
	EC	13.28 ± 0.03	1.33 ± 0.05	1.31 ± 0.03	51.92 ± 2.32	5.47 ± 0.17	18.05 ± 0.53	628.75 ± 23.51	5.12 ± 0.25	33.37 ± 0.62	2.99 ± 0.07
	Year	***	***	***	***	***	***	***	*	***	***
ANOVA	CO_2_	***	***	***	***	***	***	***	ns	***	***
	CO_2_× year	***	***	ns	***	ns	*	ns	ns	ns	*

**Table 3:** Effect of elevated CO_2_ on grain protein, starch and oil accumulation in broomcorn millet per m^2^ plot surface area.

Year	Treatment	Protein	Starch	Oil
		(g m^−2^)		
2013	CK	35.09 ± 1.54	163.85 ± 4.44	8.81 ± 0.60
	EC	33.22 ± 1.84	165.99 ± 5.39	14.40 ± 1.05
2015	CK	50.31 ± 1.52	235.12 ± 1.31	11.71 ± 2.17
	EC	56.89 ± 0.45	281.98 ± 0.54	11.95 ± 0.27
2016	CK	65.58 ± 1.09	287.93 ± 0.33	12.35 ± 0.14
	EC	83.02 ± 0.18	373.27 ± 0.34	14.77 ± 0.00
	Year	***	***	ns
ANOVA	CO_2_	***	***	**
	CO_2_× year	***	***	ns

### Metabolic changes in response to *e*CO_**2**_

**Figure 2:**
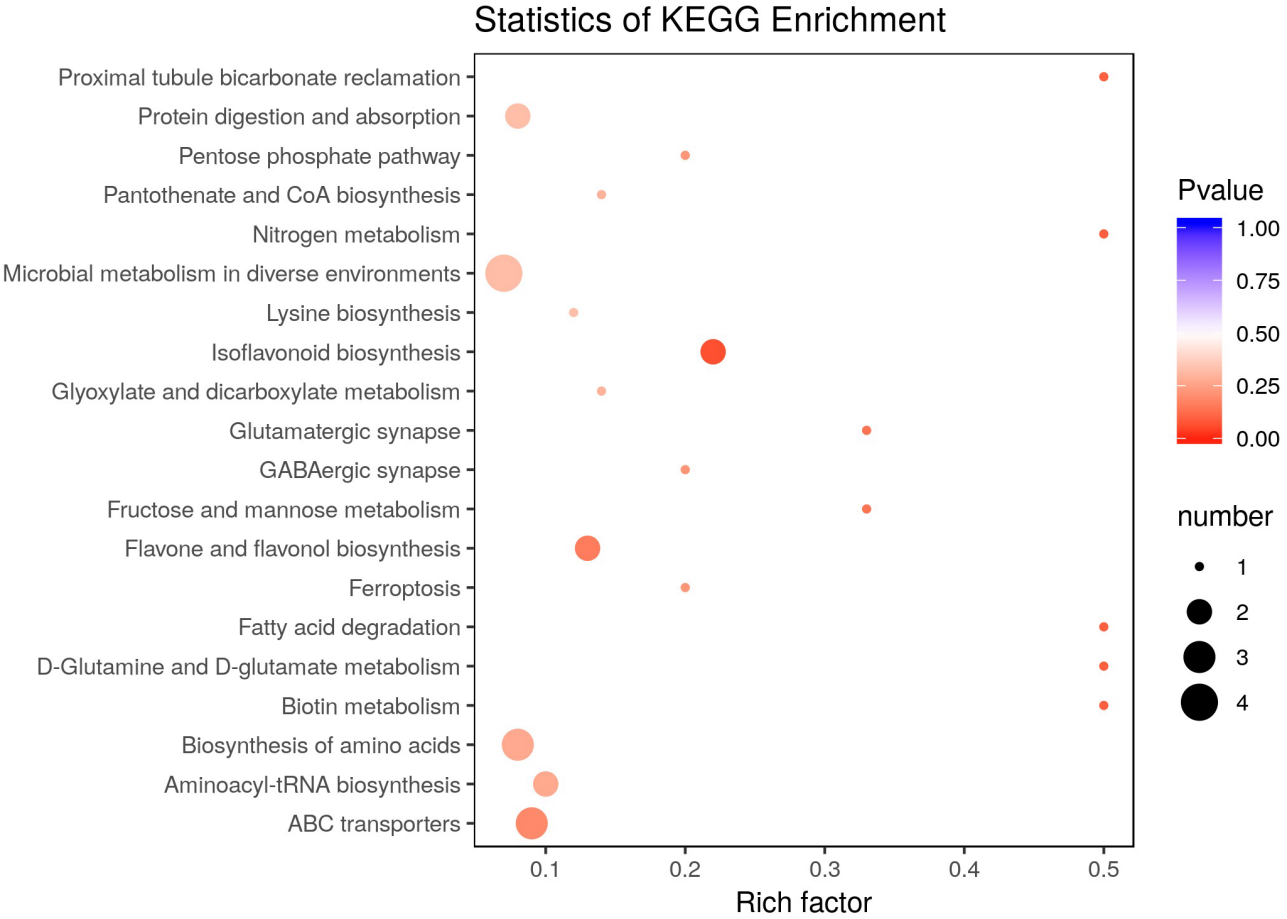
Effect of elevated CO_2_ on KEGG pathways enrichment. The Rich Factor is the ratio of the number of differentially expressed metabolites in the corresponding pathway to the total number of metabolites detected and annotated in the pathway. The color of the point is *P* value, and the redder it is, the more significant the enrichment is. The size of the point represents the number of differential metabolites enriched.

**Figure 3:**
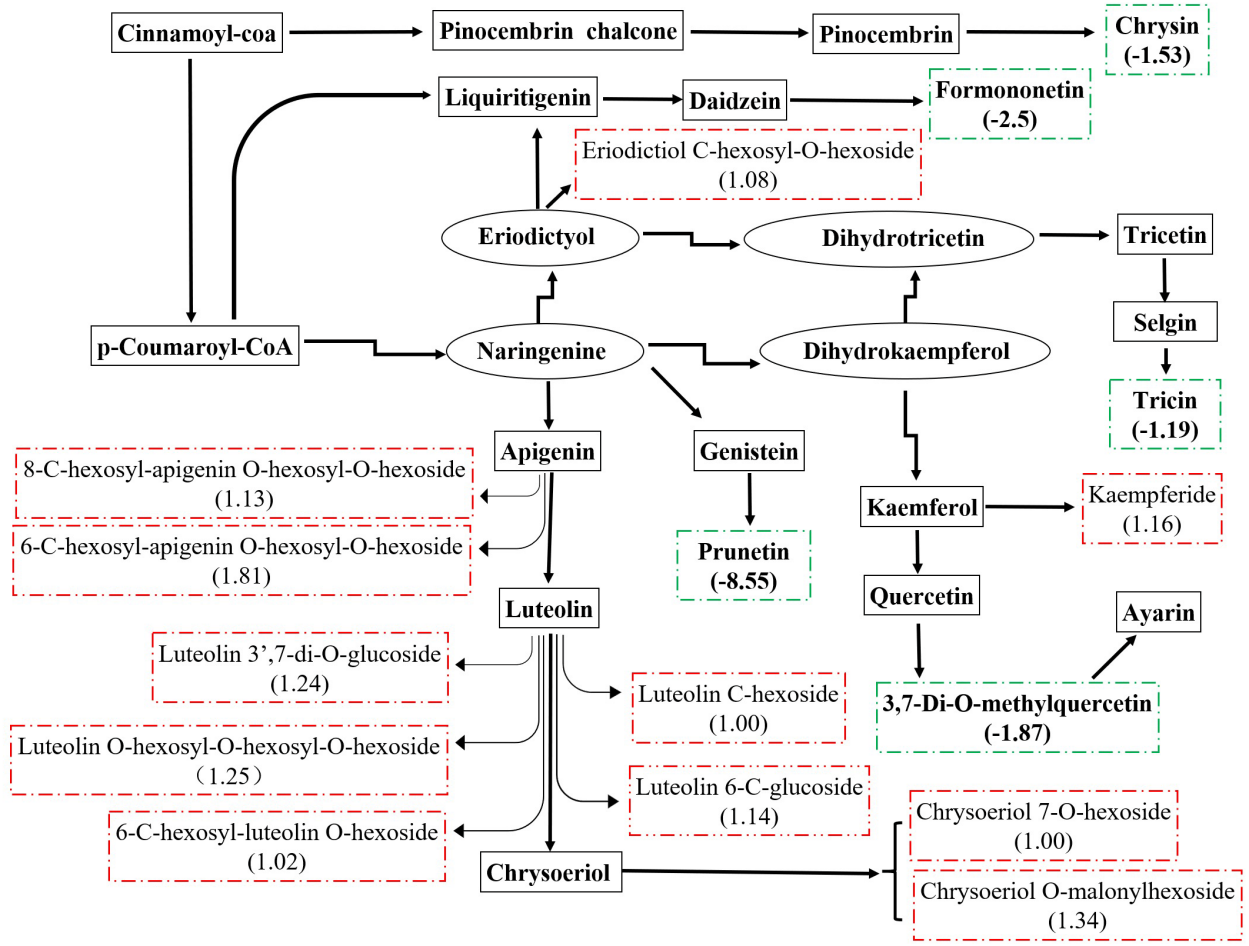
Metabolic changes in flavonoid subnetwork of broomcorn millet. The green lines represent the down-regulated metabolite. The red is up-regulated metabolite.

There were 36 metabolites with significant change under *e*CO_2_, among which 18 metabolites were increased and the others were reduced (Table 4). According to KEGG pathways analysis, *e*CO_2_ affected the synthesis of some important amino acids such as aminoacyl-tRNA biosynthesis, D-glutamine and D-glutamate metabolism, lysine biosynthesis and also affected protein digestion and absorption (Fig. 2). Elevated CO_2_ affected not only carbon metabolism (pentose phosphate pathway, glyoxylate and dicarboxylate metabolism) but also N metabolism (Fig. 2). Gluconic acid, pantothenol, and D-Mannitol all increased under *e*CO_2_ (Table 4). Flavone and flavanol biosynthesis were also affected by *e*CO_2_. Most flavonoids metabolites including eriodictyol, apigenin, luteolin, chrysoeriol, kaempferide were substantially accumulated under *e*CO_2_ (Fig. 3).

**Table 4:** Effect of elevated CO_2_ on fold changes of major metabolites in broomcorn millet.

Index	Compounds	Class	LogFC
pma1116	Kaempferide	Flavonol	1.16
pma6496	Luteolin 6-C-glucoside	Flavone C-glycosides	1.14
pmb0503	N-(4′-O-glycosyl)-p-coumaroyl agmatine	Phenolamides	1.62
pmb0588	Luteolin 3′,7-di-O-glucoside	Flavone	1.24
pmb0607	Chrysoeriol 7-O-hexoside	Flavone	1.00
pmb0608	Chrysoeriol O-malonylhexoside	Flavone	1.34
pmb0624	6-C-hexosyl-luteolin O-hexoside	Flavone C-glycosides	1.02
pmb0626	6-C-hexosyl-apigenin O-hexosyl-O-hexoside	Flavone C-glycosides	1.81
pmb0628	Eriodictiol C-hexosyl-O-hexoside	Flavone C-glycosides	1.08
pmb0639	8-C-hexosyl-apigenin O-hexosyl-O-hexoside	Flavone C-glycosides	1.13
pmb2954	Luteolin O-hexosyl-O-hexosyl-O-hexoside	Flavone	1.25
pmb3024	Luteolin C-hexoside	Flavone C-glycosides	1.00
pmb3061	5-O-p-coumaroyl quinic acid O-hexoside	Quinate and its derivatives	1.95
pmb3064	3-O-p-coumaroyl quinic acid O-hexoside	Quinate and its derivatives	2.03
pme0113	*γ*-Glu-Cys	Amino acid derivatives	1.51
pme0534	Gluconic acid	Carbohydrates	1.40
pme1261	Pantothenol	Alcohols and polyols	1.68
pme1944	D-Mannitol	Alcohols and polyols	1.15
pmb0069	Benzamide	Others	−1.07
pmb0770	N-Feruloyl serotonin	Tryptamine derivatives	−3.35
pmb0771	N-Feruloyl tyramine	Phenolamides	−3.72
pmb2653	D(+)-Melezitose O-rhamnoside	Carbohydrates	−2.38
pmb2850	Tricin	Flavone	−1.19
pmb2855	L-Glutamine O-hexside	Amino acid derivatives	−1.10
pmb2873	3-(2-Naphthyl)-D-alanine	Amino acid derivatives	−1.49
pmc1990	4′-Hydroxy-5,7-dimethoxyflavanone	Flavanone	−1.09
pme0026	L-(+)-Lysine	Amino acids	−1.44
pme0324	Chrysin	Flavone	−1.53
pme1408	L-Glutamine	Amino acids	−1.24
pme1496	Formononetin (4′-O-methyldaidzein)	Isoflavone	−2.25
pme1502	Kumatakenin	Flavonol	−1.07
pme2773	L-Cystathionine	Amino acid derivatives	−1.39
pme2827	Palmitaldehyde	Lipids_Fatty acids	−1.16
pme3288	3,7-Di-O-methylquercetin	Flavonol	−1.87
pme3292	Prunetin	Isoflavone	−8.55
pme3459	Caffeyl alcohol	Hydroxycinnamoyl derivatives	−2.07

Among the reduction of 18 metabolites, L-Glutamine and L-Lysine both involved in many pathways such as aminoacyl-tRNA biosynthesis, biosynthesis of amino acids, protein digestion and absorption, microbial metabolism in diverse environments and biosynthesis of plant secondary metabolites (Table 5). L-Glutamine participated in D-Glutamine and D-glutamate metabolism, mineral absorption, nitrogen metabolism etc.

## Discussion

### Elevated CO_**2**_ induced the increase in yields in broomcorn millet

There is broad consensus that *e*CO_2_-induced increase in the yields of C_3_ crops is higher than that of C_4_ crops. However, both our previous work (yield increased by 31.4% in 2013, (Hao et al., 2017) and the present study illustrated that the yield of broomcorn millet were significantly enhanced by *e*CO_2_ (13.6–34.2%), and the yields of foxtail millet increased on average by 21.5% in two years (Li et al., 2019). This was in contrast to the common belief that C_4_ crops (maize and sorghum) did not show yield response to *e*CO_2_, because the photosynthesis of C_4_ crops was saturated at current atmospheric (CO_2_) (Leakey et al., 2006; Markelz, Strellner & Leakey, 2011). While, our previous work proved that photosynthetic rate and intrinsic water use efficiency of broomcorn millet were significantly increased under *e*CO_2_ (Hao et al., 2017; Zhang et al., 2020). Similar responses of millets were observed by Sage & Zhu (2011). This may be conducive to an increase in yield of broomcorn millet. This study further suggested that *e*CO_2_ affected pentose phosphate pathway, glyoxylate and dicarboxylate metabolism, fructose and mannose metabolism, which in turn increased accumulation of carbohydrates such as glucose acid, leading to an increase in grain yield of broomcorn millet.

**Table 5:** The downregulation of pme0026 and pme1408 under elevated CO_2_ compared to ambient CO_2_ involved in pathways in broomcorn millet.

Pathway	pme0026 L-Glutamine	pme1408 L-Lysine
2-Oxocarboxylic acid metabolism		√
ABC transporters	√	√
Alanine, aspartate and glutamate metabolism	√	
Aminoacyl-tRNA biosynthesis	√	√
Arginine biosynthesis	√	
Biosynthesis of alkaloids derived from ornithine, lysine and nicotinic acid		√
Biosynthesis of amino acids	√	√
Biosynthesis of antibiotics		√
Biosynthesis of plant secondary metabolites	√	√
Biosynthesis of secondary metabolites		√
Biotin metabolism		√
D-Glutamine and D-glutamate metabolism	√	
GABAergic synapse	√	
Glutamatergic synapse	√	
Glyoxylate and dicarboxylate metabolism	√	
Lysine biosynthesis		√
Lysine degradation		√
Metabolic pathways	√	√
Microbial metabolism in diverse environments	√	√
Mineral absorption	√	
Nitrogen metabolism	√	
Protein digestion and absorption	√	√
Proximal tubule bicarbonate reclamation	√	
Purine metabolism	√	
Pyrimidine metabolism	√	
Tropane, piperidine and pyridine alkaloid biosynthesis		√
Two-component system	√	

### Effect of *e*CO_**2**_ on grain concentration and accumulations of protein, starch and oil

Broomcorn millet had a higher protein content more than 11% (dry basis), which was higher than the 7.2% protein found in rice and 10.4% found in sorghum (Jha et al., 2013). The protein quality of broomcorn millet (Essential Amino Acid Index) was higher (51%) compared to wheat (Kalinova & Moudry, 2006). Elevated CO_2_ reduced N concentrations by 4%–21% in non-legumes such as wheat, cotton and sorghum at elevated CO_2_ (540–958 µmol mol^−1^) (Prior et al., 1994), while it was reduced by only 1.5%-−2.1% in legumes (Lam et al., 2012). The effect of *e*CO_2_ on the protein concentration of C_4_ crops was less studied. Our study suggested that *e*CO_2_ significantly reduced the protein concentration of broomcorn millet by 7.8% over an average of three years. Elevated CO_2_ decreased N concentrations in plants through: (1) increasing plant biomass, the so-called ‘dilution effect’ (Reich et al., 2006); (2) reducing the prevalent protein Rubisco concentrations (Long et al., 2004); (3) changing the rhizosphere environment and limiting the N available for plant uptake (Reich et al., 2018); and (4) inhibiting plant N metabolism, especially the assimilation of nitrate of C_3_ plants (Butterly et al., 2015). Elevated CO_2_-induced inhibition of N uptake and assimilation would decrease organic N production and retard growth of C_3_ plants (Bloom et al., 2012). In the present study, *e*CO_2_ also affected the grain N metabolism of broomcorn millet (Fig. 2). At the same time, *e*CO_2_ affected synthesis of some important amino acids such as aminoacyl-tRNA biosynthesis, D-glutamine and D-glutamate metabolism, lysine biosynthesis and also affected protein digestion and absorption, thus influencing the protein content (Fig. 2). In addition, *e*CO_2_ had reduced the specific nitrogenous metabolites (L-lysine metabolites and L-glutamine), which were the most represented amino acid in grain proteins. The fact that lysine recycling was perturbed by *e*CO_2_ suggested that sustain amino acid metabolism and protein synthesis was inhibited. It was also reported that a typical decrease in glutamine under *e*CO_2_ in wheat grain (Soba et al., 2019; Högy et al., 2009) and soybean seed (Li & Siddique, 2018). Some studies revealed that glutamine had been reported to accumulate under *e*CO_2_ (Yu et al., 2012; Watanabe et al., 2014; AlJaouni et al., 2018). Hence, impact of *e*CO_2_ on glutamine was expected to show some kind of complexity. The effects of *e*CO_2_ on metabolites were different among species. The decrease in protein was also caused by the carbon dilution effect due to the significant increase in grain yield for three years. Elevated CO_2_ significantly increased protein accumulation due to the significant increase in grain yield except in 2013. Jobe et al. (2020) revealed that C_4_ plants required less total N, had higher N use efficiency, and maintain N levels under *e*CO_2_.

In our study, *e*CO_2_ had no significant effect on the grain oil concentration of broomcorn millet, while significantly increased the grain oil accumulation as a result of the significant increase in grain yield. Elevated CO_2_ had been shown to increase oil concentrations in soybean seed (Hao et al., 2014; Li & Siddique, 2018) and oilseed rape (Högy et al., 2010). Enhanced oil synthesis and storage in plants under *e*CO_2_ had been linked to carbon and energy supply (Rawsthorne, 2002; Bates, Stymne & Ohlrogge, 2013). Singh et al. (2016) also stated that the increase in seed oil concentration under *e*CO_2_ was attributed to the enhancement of photosynthesis. Starch was the major component in proso millet grain, accounting for 58.1%–77.9% of the total grain weight (Yang et al., 2018). In our study, the effect of *e*CO_2_ on the starch content and starch accumulation of broomcorn millet was similar to that of oil, which also proved that starch quality was improved under *e*CO_2_. Mao et al. (2020) also reported that *e*CO_2_ alone also had no significant effects on starch content in rice grains, which is consistent with our findings.

### Effect of *e*CO_**2**_ on grain mineral quality

Dietary deficiency of Zn and Fe is a major global public health problem. Myers et al. (2015) predicted the risk of global Zn deficiency would increase under *e*CO_2_. Their meta-analysis results demonstrated that *e*CO_2_ significantly decreased the concentrations of Zn and Fe in all C_3_ grasses and legumes investigated, but not for C_4_ crops (maize and sorghum) (Myers et al., 2014). Our research also confirmed that *e*CO_2_ had less effect on Zn and Fe concentrations of C_4_ millet, suggesting that C_4_ crops had more desirable nutritional traits for combating hidden hunger. Millets are considered as high-energy yielding nourishing foods which help in addressing malnutrition (Nithiyanantham et al., 2019). Consumption of broomcorn millet is associated with reduced risk of type-2 diabetes mellitus because whole grains are a rich source of Mg (Pathak, 2013). Mg acts as a co-factor in a number of enzymatic reactions that regulate the secretion of glucose and insulin. Mg can also reduce the frequency of migraine headaches and heart attacks, thus it is beneficial for people suffering from atherosclerosis and diabetic heart disease (Shobana & Malleshi, 2007; Gélinas et al., 2008). Elevated CO_2_ significantly increased grain Mg concentration across the three years on average by 27.3%. This exceptional nutritional properties of broomcorn millet make it an excellent crop, essential for food, nutrition, and health security under climate change scenarios. We also observed a significant increase in the concentration of Mn in broomcorn millet. In contrast, *e*CO_2_ decreased the Mn content in nine diverse rice cultivars by 53% (Ujiie et al., 2019). A previous study revealed that consumption of a Mn-deficient diet (0.11 mg/day; about 3% of RDA) for 39 days caused a mild form of dermatitis among five of seven men (Friedman et al., 1987). Boron was involved in enzyme reactions (Seth & Aery, 2017), as well as in photosynthesis and carbohydrate metabolism (Han et al., 2008; Han et al., 2009; Teija et al., 2011), flavonoid synthesis, and nitrogen (Camacho-Cristóbal & González-Fontes, 2007) and phenol metabolism (Teija et al., 2011). A significant increase in B was observed in broomcorn millet under *e*CO_2_. Carbohydrate dilution (Reich et al., 2006) and decreased mass flow due to reduced transpiration (McGrath & Lobell, 2013; Houshmandfar et al., 2018) may all be relevant to explain this phenomenon of decreased grain mineral concentration value under *e*CO_2_. The carbon dilution effect due to the significant increase in grain yield across the three years did not occur in Mn, Mg, and B. In our previous study, transpiration rate in broomcorn millet was increased by an average of 12.0% and 21.2% in 2013 and 2014, respectively (Hao et al., 2017). Increases in transpiration may stimulate translocation of nutrients from the root to shoot, and then to the grain of broomcorn millet. Pérez-López et al. (2014) suggested that the increase in growth under *e*CO_2_ could be attributed to the stimulation of metabolic activity in plants, and, accordingly, to the requirement for nutrients that serve as enzyme cofactors in metabolic reactions (Mg and Mn). In the present study, the increase in concentration of Mn, Mg, and B of broomcorn millet observed in response to *e*CO_2_ suggests improved nutritional value of soybean under the scenario of elevated CO_2_ concentration. The influence of *e*CO_2_ on elemental content varied among years, possibly because the elemental content of grains was affected by environmental factors during the ripening period, such as air temperature (Seo & Chamura, 1980). While no significant changes were found in the concentrations of K, Ca, Cu and Zn under *e*CO_2_ on average for three years in broomcorn millet, similar to the findings of Myers et al. (2014). This confirmed that increase in atmospheric (CO_2_) would have less effect on nutrient content of C_4_ crops. Grain Zn accumulation were increased by *e*CO_2_ in the present, in agreement with this study (Fernando et al., 2012). The increase in the grain mineral accumulations of K, Mn, Ca, Mg, Zn, B for three years might be explained by the stronger increase (34.2%) in grain yield in 2013 (Hao et al., 2017), compared with the increase of 19.4% and 29.9% in 2015 and 2016, respectively. Thus, besides possibly decreased nutrient concentrations, increased grain nutrient accumulations should be considered in assessing the impacts of future *e* CO_2_ on human nutrition. As said in Jobe et al. (2020), broomcorn millet, as an orphan crop, has great potential to deliver sufficient nutrients for human food and health. More effort is needed to understand the control of nutrient fluxes and homeostasis in broomcorn millet to ensure that this will also be true in the coming decades.

### Effect of *e*CO_**2**_ on flavonoids

Millet grain was abundant in health-beneficial phenolic compounds compromise phenolic acids, flavonoids, and tannins, which were beneficial to human health (Hassan, Sebola & Mabelebele, 2021). Millets flavonoids had shown a remarkable spectrum of therapeutic properties for medical and clinical applications, such as anti-inflammatory, anti-cancer, antihy- pertensive, diuretic, analgesic, and hypolipidemic effects (Banerjee et al., 2012; Chethan, 2008; Edge, Jones & Marquart, 2005; Ekta & Sarita, 2016; Li et al., 2022). A recent study revealed that the contents and types of flavonoids compounds in minor grains (sorghum, foxtail millet, and broomcorn millet) were more than those in staple crops (wheat, maize, and rice) (Tang et al., 2022). Kaempferol and apigenin were major flavonoids in raw millets (Pradeep & Sreerama, 2015). In this study, they were substantially accumulated under *e*CO_2_. In addition, eriodictyol, luteolin, and chrysoeriol were also accumulated under *e*CO_2_. Weisshaar & Jenkins (1998) stated that approximately 20% of the carbon from photosynthesis was used to synthesize the phenolic compounds found in nature, including flavonoids. Many studies have shown that *e*CO_2_ significantly stimulates plant growth and improves photosynthetic products of plants (Li et al., 2017; Gong et al., 2021), thus increasing precursors of flavonoids, and consequently, increasing the flavonoid content in plants (Karimi, Jaafar & Ghasemzadeh, 2016; Sgherri et al., 2017; Hozzein et al., 2020; Zhang et al., 2021). The previous study revealed that CO_2_ enrichment lead to increased photosynthesis rates in broomcorn millet (Hao et al., 2017). Therefore, larger amounts of carbon could be obtained to generate flavonoids from broomcorn millet under *e*CO_2_. In the present study, the increase in flavonoids of broomcorn millet in response to *e*CO_2_ indicated that the nutritional value of broomcorn millet was improved under the scenario of *e*CO_2_. Therefore, broomcorn millet could be used as nutritional supplements in the area of therapy. Similarly, *e*CO_2_ enhanced the production of flavonoids in soybean (Li & Siddique, 2018; O’Neill et al., 2010; Kretzschmar et al., 2009), while it decreased the total flavonoid content in rice grain (Goufo et al., 2014). These studies suggested that *e*CO_2_-induced flavonoid changes exhibited some degree of variability, depending on the plant species.

Cultivating broomcorn millet promotes access to better nutrition for communities, especially in the developing regions of the world. Food security can be further improved by diversifying our staple foods to provide more options for combating climate change. The United Nations Food and Agriculture Organization has announced the year 2023 as ‘International Year of Millets’ (https://news.un.org/en/story/2021/05/1092492), recognizing the potential of this crop. By that time, it may be expected to incentivize increased millet production and achieve success in combating hunger and malnutrition among the vulnerable population in any future aberrant conditions.

## Conclusions

Elevated CO_2_ significantly increased the yield of broomcorn millet. The concentration of K, Ca, Cu and Zn in broomcorn millet was not changed under *e*CO_2_, whereas the concentration of Mn, Mn, and B was increased. A majority of antioxidant metabolite (flavonoids) tended to accumulate under *e*CO_2_. The protein content was significantly decreased, whereas starch concentration and oil concentrations were not affected by *e*CO_2_. The increase in grain mineral accumulations of K, Mn, Ca, Mg, Zn, B, grain starch accumulation and oil accumulation across the three years was accompanied by the stronger grain yield increase. The negative effects of *e*CO_2_ on nutrient quality in broomcorn millet were smaller. Broomcorn millet have more desirable nutritional traits for combating hidden hunger. This may potentially be useful for breeding more nutritious plants in the era of climate change.

##  Supplemental Information

10.7717/peerj.14024/supp-1 Supplemental Information 1 Raw data of yield, grain mineral, protein, starch and oil concentrations and accumulation

## Additional Information and Declarations

### Data Availability

The following information was supplied regarding data availability: The raw data is available in the Supplemental Files.
